# Correction: Climate change in Bangladesh: Temperature and rainfall climatology of Bangladesh for 1949–2013 and its implication on rice yield

**DOI:** 10.1371/journal.pone.0295718

**Published:** 2023-12-06

**Authors:** Edris Alam, Al-Ekram Elahee Hridoy, Shekh Md. Shajid Hasan Tusher, Abu Reza Md. Towfiqul Islam, Md Kamrul Islam

There is error in the Funding statement. The correct Funding statement is as follows: This work was financially supported by the Deanship of Scientific Research at the King Faisal University, Saudi Arabia (grant: 3,690).

The affiliation for the third author is incorrect. Shekh Md. Shajid Hasan Tusher is not affiliated with #2 but with #1: Department of Geography and Environmental Studies, University of Chittagong, Chittagong, Bangladesh.
