# Supplementary material for: Climate change in Bangladesh: Temperature and rainfall climatology of Bangladesh for 1949–2013 and its implication on rice yield
Source: PLoS One. 2023 Oct 12;18(10):e0292668. doi: 10.1371/journal.pone.0292668 (PMC10569571; doi:10.1371/journal.pone.0292668)
Supplement: Attachment — Submitted filename: Responses to reviewers letter 8 Sept 2023.docx [file pone.0292668.s001.docx]

Date: 27 August 2023

Dear Professor Md. Naimur Rahman,

RE: Revision to Manuscript ID: PONE-D-23-16309 entitled “Climate Change in Bangladesh: Temperature and Rainfall Climatology of Bangladesh for 1949–2013 and its implication on rice yield” by Alam

On behalf of the authorship of this paper, I would like to thank the Editor and Referees for the review of the original submission and the resubmission that includes a set of very helpful and constructive comments. We have reworked the paper in response to these. A description of the responses to each comment is detailed in the Table below indicating how modifications have been made within the paper. In a very few instances, we have chosen not to follow an individual reviewer suggestion, though provide a detailed clarification where this is the case. I trust this revised manuscript meets all of the standards of the *Plos One Journal* and am ready to respond to any further points that would arise.

Yours sincerely,

Dr. Edris Alam

Table detailing our responses to the Reviewer comments point-by-point.

| **Comments of the reviewers** | **My responses** | **Place in revised manuscript where response is detailed** |
| --- | --- | --- |
| **Reviewer ≠ 1** | | |
| **General comments** | | |
| General comments The study focuses on temperature and rainfall climatology and implication on rice yield. Though the objective of the study is to determine temperature and rainfall climatology implication on rice yield for time series for longer period. The author tried to make the paper comprehensive, but it still requires some major tasks to be completed for improvement of its various sections. | We are grateful to the reviewer for this assessment. By following the reviewer suggestion, we have improved various sections of the manuscript. | - |
| Abstract 1. The abstract is informative up to the mark. It describes fully to reflect the whole study. | We are grateful to the reviewer for the positive appraisal of the paper and recognizing the significance of this study. | - |
| Introduction 1. The introduction seems over explanatory; it should be more concise.  2. Line 63: Citation of IPCC 2001 should be updated. I believe IPCC published more exclusive reports regarding the issue after 2001 (ex. Line 65)  3. What is the evidence of this statement in Line 141? | We have concise the introduction and removed unnecessary information from the text  We have updated this in the revised introduction section.  We have provided the evidence of this statement in revised version | -  Lines 51, 58 and 66  Lines 120-121 |
| Methodology 1. Overall, analytical techniques are well described in a scientific way and well fitted to the study.  2. Why the data duration is from 1949-2013? The decadal temperature changes in results and discussion section. So, did not temperature change in between 2013 to 2023? It is not enough to describe as limitation of this study. It could add more value to this study and most importantly the results could be different. Because, rice yield area (Table-2 of https://journals.plos.org/plosone/article?id=10.1371/journal.pone.0261128), production (Table-3 of https://journals.plos.org/plosone/article?id=10.1371/journal.pone.0261128) has been changed between 2008-2020.  3. It is advised to add data from 2013- till the available date (2023 or 2022) in this study and analyze again to get more specific results. | We are grateful to the reviewer for the positive appraisal of the method section.  Respectfully, this suggestion is not out of place. This is main drawback of this work. The novel aspect is that we have considered long-term consistent 65-years dataset. We have mentioned this main drawback in the discussion section. We have also provided a justification in method section.  We have already responded to this suggestion | - |
| Results 1. The presentation of results is well organized.  2. According to methodology, results are well described.  3. After adding data from 2013-2023 (2022), please write results accordingly. | We are grateful to the reviewer for this assessment.  We are grateful to the reviewer for this assessment.  We have already responded to this suggestion  . | - |
| Discussion 1. The discussion is also well described.  2. Line 561: Please mention some recent major studies with example. If the studies did not use longer time series data, please show the comparison of the data periods. From line 533-560 author showed comparison of results with other studies, not data sets.  3. After adding data from 2013-20233 (2022), please write discussion accordingly. | We are grateful to the reviewer for positive appraisal.  We have already responded to this suggestion | - |
| Conclusion 1. The revised conclusion should be based on the revised methodology. | We have revised the conclusion section | Lines 641-644  Lines 655-664 |
| **Reviewer #2** |  |  |
| General comments | | |
| The manuscript has some shortcomings which need to be improved prior to its publication. My recommendation is that the article needs major revisions before it can be considered for publication. | We are grateful to the reviewer for this assessment. By following the reviewer suggestion, we have improved various sections of the manuscript. |  |
| 1. Abstract: The abstract is a bit generic. Please add some more information regarding your results. It should be improved in a quantitative way. | Respectfully, further addition in the abstract would exceed word limit for this section. |  |
| 2. Author should specify the key objectives of this research work in last paragraph of introduction section. | We have accepted this suggestion and have presented the key objectives of this research work in last paragraph of introduction section. |  |
| 3. Introduction is generalized. I would recommend following recent research articles to reconstruct this section with extensive literature “Profitable agricultural land use planning in a red and lateritic soil of subtropical environment using field-based index of crop suitability (ICS” “Field based index of land suitability (ILS): a new approach for rainfed paddy crop production in groundwater scarce region” | We have accepted this suggestion. We have followed and used the suggested articles to reconstruct the introduction of this paper. |  |
| 4. Methodology section is weakly written. So, my suggestion is to reconstruct it. Author need to review more about RS-GIS environment and current techniques. “Characterization of groundwater potential zones in water-scarce hardrock regions using data driven model” “Hydrogeochemical Evaluation of Groundwater Aquifers and Associated Health Hazard Risk Mapping Using Ensemble Data Driven Model in a Water Scares Plateau Region of Eastern India” “Hydrogeochemical evaluation and corresponding health risk from elevated arsenic and fluoride contamination in recurrent coastal multi-aquifers of eastern India”  “Application of novel data-mining technique based nitrate concentration susceptibility prediction approach for coastal aquifers in India” “Hydro-chemical assessment of groundwater pollutant and corresponding health risk in the Ganges delta, Indo-Bangladesh region” Hydrogeochemical characterization based water resources vulnerability assessment in India's first Ramsar site of Chilka lake | We recognize this suggestion and have incorporated the following articles to improve the method section. |  |
| 5. Discussion section should be written by comparing with already published articles in this concept. | We have accepted this suggestion and have further improved the discussion section |  |
| 6. In conclusion section, you have to mention the implications of your research and how it makes a footprint in scientific research. Try to incorporate your work to global interest how this research has worldwide importance. It will be interesting for the readers. | This suggestion coincides with the suggestion of the first reviewer and we have already improved this section. | Lines 648-651  Lines 662-671 |
| 7. Reference: Re-check the whole reference just to make sure you have added all the references that you cited in your manuscript. | We have accepted this suggestion and have rechecked the entire reference list |  |
| 8. Apart from this the quality of the overall paper is very good. I prefer this article with acceptable with major modifications. | We are grateful to the reviewer for this assessment. We have considered constructive feedback at our best. |  |
| Editorial feedbacks | | |
| 1. Please ensure that your manuscript meets PLOS ONE's style requirements, including those for file naming. The PLOS ONE style templates can be found at  https://journals.plos.org/plosone/s/file?id=wjVg/PLOSOne_formatting_sample_main_body.pdf and  https://journals.plos.org/plosone/s/file?id=ba62/PLOSOne_formatting_sample_title_authors_affiliations.pdf | We recognize this suggestion and have used template and maintained authors guidelines |  |
| 2. Thank you for stating the following financial disclosure:  “The funders had no role in study design, data collection and analysis, decision to publish, or preparation of the manuscript.”  At this time, please address the following queries:  a) Please clarify the sources of funding (financial or material support) for your study. List the grants or organizations that supported your study, including funding received from your institution.  b) State what role the funders took in the study. If the funders had no role in your study, please state: “The funders had no role in study design, data collection and analysis, decision to publish, or preparation of the manuscript.”  c) If any authors received a salary from any of your funders, please state which authors and which funders.  d) If you did not receive any funding for this study, please state: “The authors received no specific funding for this work.”  Please include your amended statements within your cover letter; we will change the online submission form on your behalf. | We recognize this suggestion and stated the following: The authors received no specific funding for this work |  |
| 3. We note that Figures 1-3 in your submission contain [map/satellite] images which may be copyrighted. All PLOS content is published under the Creative Commons Attribution License (CC BY 4.0), which means that the manuscript, images, and Supporting Information files will be freely available online, and any third party is permitted to access, download, copy, distribute, and use these materials in any way, even commercially, with proper attribution. For these reasons, we cannot publish previously copyrighted maps or satellite images created using proprietary data, such as Google software (Google Maps, Street View, and Earth). For more information, see our copyright guidelines: http://journals.plos.org/plosone/s/licenses-and-copyright.  We require you to either (1) present written permission from the copyright holder to publish these figures specifically under the CC BY 4.0 license, or (2) remove the figures from your submission:  a. You may seek permission from the original copyright holder of Figures 1-3 to publish the content specifically under the CC BY 4.0 license.  We recommend that you contact the original copyright holder with the Content Permission Form (http://journals.plos.org/plosone/s/file?id=7c09/content-permission-form.pdf) and the following text:  “I request permission for the open-access journal PLOS ONE to publish XXX under the Creative Commons Attribution License (CCAL) CC BY 4.0 (http://creativecommons.org/licenses/by/4.0/). Please be aware that this license allows unrestricted use and distribution, even commercially, by third parties. Please reply and provide explicit written permission to publish XXX under a CC BY license and complete the attached form.”  Please upload the completed Content Permission Form or other proof of granted permissions as an "Other" file with your submission.  In the figure caption of the copyrighted figure, please include the following text: “Reprinted from [ref] under a CC BY license, with permission from [name of publisher], original copyright [original copyright year].”  b. If you are unable to obtain permission from the original copyright holder to publish these figures under the CC BY 4.0 license or if the copyright holder’s requirements are incompatible with the CC BY 4.0 license, please either i) remove the figure or ii) supply a replacement figure that complies with the CC BY 4.0 license. Please check copyright information on all replacement figures and update the figure caption with source information. If applicable, please specify in the figure caption text when a figure is similar but not identical to the original image and is therefore for illustrative purposes only.  The following resources for replacing copyrighted map figures may be helpful:  USGS National Map Viewer (public domain): http://viewer.nationalmap.gov/viewer/  The Gateway to Astronaut Photography of Earth (public domain): http://eol.jsc.nasa.gov/sseop/clickmap/  Maps at the CIA (public domain): https://www.cia.gov/library/publications/the-world-factbook/index.html and https://www.cia.gov/library/publications/cia-maps-publications/index.html  NASA Earth Observatory (public domain): http://earthobservatory.nasa.gov/  Landsat: http://landsat.visibleearth.nasa.gov/  USGS EROS (Earth Resources Observatory and Science (EROS) Center) (public domain): http://eros.usgs.gov/#  Natural Earth (public domain): http://www.naturalearthdata.com/ | Respectfully, we confirm that we have produced all figures used in this paper. There is no copyright issue involved with any figures. |  |
| **Additional Changes to the revised manuscript** | | |
| Manuscript text | Having had the opportunity to revise our manuscript, I have made significant efforts to shorten the text and improve the quality of expression throughout. | Various locations throughout the revised text |

I cannot thank both the editor and reviewer enough for their generous, positive commentary and general support for this work. I do believe this revised version of the manuscript is substantially improved.

I trust that these responses are acceptable and look forward to hearing from you in due course.

Yours sincerely,
